# ETS-targeted therapy: can it substitute for MEK inhibitors?

**DOI:** 10.1186/s40169-017-0147-4

**Published:** 2017-05-02

**Authors:** Osamu Tetsu, Frank McCormick

**Affiliations:** 1 Department of Otolaryngology-Head and Neck Surgery, School of Medicine, University of California, San Francisco, San Francisco, CA 94143 USA; 2 UCSF Helen Diller Family Comprehensive Cancer Center, School of Medicine, University of California, San Francisco, San Francisco, CA 94143 USA

**Keywords:** ETS1/2, Targeted therapy, MEK inhibitors, Adaptive drug resistance, Proteolysis, c-Src, USP9X, Transcriptional co-activators, CBP/p300, BRD4

## Abstract

**Background:**

The RAS/MAPK pathway has been intensively studied in cancer. Constitutive activation of ERK1 and ERK2 is frequently found in cancer cells from a variety of tissues. In clinical practice and clinical trials, small molecules targeting receptor tyrosine kinases or components in the MAPK cascade are used for treatment. MEK1 and MEK2 are ideal targets because these enzymes are physiologically important and have narrow substrate specificities and distinctive structural characteristics. Despite success in pre-clinical testing, only two MEK inhibitors, trametinib and cobimetinib, have been approved, both for treatment of BRAF-mutant melanoma. Surprisingly, the efficacy of MEK inhibitors in other tumors has been disappointing. These facts suggest the need for a different approach. We here consider transcription factor ETS1 and ETS2 as alternate therapeutic targets because they are major MAPK downstream effectors.

**Main text:**

The lack of clinical efficacy of MEK inhibitors is attributed mostly to a subsequent loss of negative feedback regulation in the MAPK pathway. To overcome this obstacle, second-generation MEK inhibitors, so-called “feedback busters,” have been developed. However, their efficacy is still unsatisfactory in the majority of cancers. To substitute ETS-targeted therapy, therapeutic strategies to modulate the transcription factor in cancer must be considered. Chemical targeting of ETS1 for proteolysis is a promising strategy; Src and USP9X inhibitors might achieve this by accelerating ETS1 protein turnover. Targeting the ETS1 interface might have great therapeutic value because ETS1 dimerizes itself or with other transcription factors to regulate target genes. In addition, transcriptional cofactors, including CBP/p300 and BRD4, represent intriguing targets for both ETS1 and ETS2.

**Conclusions:**

ETS-targeted therapy appears to be promising. However, it may have a potential problem. It might inhibit autoregulatory negative feedback loops in the MAPK pathway, with consequent resistance to cell death by ERK1 and ERK2 activation. Further research is warranted to explore clinically applicable ways to inhibit ETS1 and ETS2.

## Introduction

**Fig. 1:**
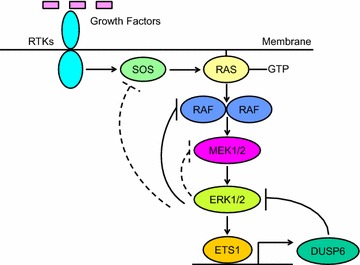
Negative feedback regulates the RAS/MAPK pathway. Although MAPK signaling was previously considered linear, autoregulatory negative feedback loops precisely control signaling output. MEK inhibitors reduce activity of ERK1 and ERK2 and then relieve the feedback inhibition of RAF, resulting in enhancement of RAF kinase activity. Likewise, ETS1-targeted therapy may activate ERK1 and ERK2 by inhibiting DUSP6, which is an ETS1 transcriptional target

Mitogen-activated protein kinases (MAPKs) are serine (Ser) or threonine (Thr) protein kinases that respond to stimulation from extracellular growth factors through specific cell surface receptors [1–4]. They are a part of major signaling cascades. Among MAPKs, extracellular signal-regulated kinases 1 and 2 (ERK1 and ERK2) have been most characterized (Fig. 1). Activated receptor tyrosine kinases (RTKs) recruit adaptor proteins and guanine nucleotide exchange factors (GEFs: SOS1 and SOS2) to trigger RAS (HRAS, KRAS, or NRAS), which drives the formation of high-activity homo- or heterodimers of RAF (also known as MAPKKK: ARAF, BRAF, or CRAF), causing phosphorylation and activation of MEK1 and MEK2 (MAPKK) with consequent ERK1 and ERK2 (classical MAPK) stimulation. Numerous proteins are identified as their substrates, among which transcription factors ETS1, ETS2, and AP-1 are particularly important [1, 4]. They regulate expression of matrix metalloproteases, BCL-2 family members, and D-type cyclins to mediate cellular invasion and migration, cell survival and anti-apoptosis, and entry into the S phase from the G1 phase in the cell cycle [5–12].

The RAS/MAPK pathway has been intensively studied [1–4], with constitutive activation of ERK1 and ERK2 found frequently in human cancer cells from a variety of tissues (e.g., lung, pancreas, colon, ovary, kidney, skin, and thyroid) [13]. Amplification, overexpression, or mutations in RTKs and genetic alterations in upstream components of the MAPK pathway, including KRAS, NRAS, HRAS, CRAF, BRAF, MEK1, and MEK2, alter cell signaling in tumors. In clinical practice and clinical trials, small molecules targeting RTKs or components in the MAPK cascade are used to treat cancer [1, 3, 4]. MEK1 and MEK2 are ideal targets; not only do they play a key role in tumor development and progression [3, 4], they have narrow substrate specificities and distinctive structural characteristics.

MEK activation through the MAPK signaling cascade is necessary for mammalian cell transformation, and constitutively active MEK mutants promote transformation of fibroblast cells [14, 15]. Furthermore, MEK inhibitors inhibit growth of xenografted human and murine colon carcinomas [16], a mechanism we have studied [11]. Treating colon cancer cells with small-molecule MEK inhibitors blocked both CDK4 and CDK2 kinase activity and arrested G1 growth. MEK1 and MEK2 also have a crucial role in inhibition of apoptosis [17, 18]: their downstream effectors ERK1 and ERK2 phosphorylate a BH3-only protein Bim at Ser69, targeting it for degradation via the proteasome-ubiquitin pathway.

ERK1 and ERK2 are the only known physiological substrates of MEK1 and MEK2 [1–4]—a narrow specificity that allows the development of MEK inhibitors with fewer off-target side effects. MEK1 and MEK2 are dual-specificity protein kinases and phosphorylate both tyrosine (Tyr) and Thr residues of ERK1 and ERK2—Tyr204 and Thr202 in ERK1 and Tyr187 and Thr185 in ERK2—rendering them active [19]. On the other hand, MEK1 and MEK2 themselves are phosphorylated and activated by RAF in two key Ser residues in the regulatory loop—Ser218 and Ser222 in MEK1 and Ser222 and Ser226 in MEK2 [20].

MEK inhibitors are divided into two groups, ATP-competitive and -noncompetitive [21], although most of the known MEK inhibitors are the latter [21]. By not competing directly for the ATP-binding site, they avoid competition with high intracellular ATP levels [22]. Structural analysis of MEK1 and MEK2 with PD184352, a putative non-competitive inhibitor, has revealed that the molecules possess a unique allosteric inhibitor-binding pocket adjacent to, but separate from, the ATP-binding site [23]. Once the MEK inhibitor binds the pocket, several conformational changes follow, causing MEK1 and MEK2 to be locked into a catalytically inactive state. These facts may provide an explanation for why this class of MEK inhibitors has shown keen specificity and high potency.

However, despite promising drug targets and success in pre-clinical testing, only two MEK inhibitors, trametinib (Mekinist) and cobimetinib (Cotellic), have been approved, both for the treatment of BRAF-mutant melanoma [3, 4, 24, 25]. The efficacy of MEK1/2 inhibitors in other tumors has been more disappointing [3, 4]. These facts suggest the need for an alternative approach. To this end, we present our perspective on ETS-targeted therapy.

### Most MEK inhibitors have limited clinical efficacy

Numerous potent, selective allosteric MEK inhibitors have been developed and have undergone clinical evaluation of their ability to inhibit tumor growth [3, 4]. Preclinical studies showed efficient inhibition of phosphorylation of ERK1 and ERK2, which correlates with potent growth inhibition in cancer cell lines with mutant BRAF or RAS with elevated phosphorylation of MEK1 and MEK2 [3, 4, 26, 27]. However, most MEK inhibitors have demonstrated limited clinical efficacy as single-agent therapies [3, 4]. Only trametinib (see above) showed improved progression-free and overall survival both as a single agent and in combination with the BRAF inhibitor, dabrafenib [28–30]. More recently, another MEK inhibitor, cobimetinib—when used in combination with the BRAF-inhibitor, vemurafenib—was reported to improve progression-free survival among patients with BRAF V600-mutated metastatic melanoma [25]. These facts underscore the challenge of bringing MEK inhibitors from bench to bedside.

### Loss of negative feedback regulation may reduce the clinical efficacy of MEK inhibitors

Loss of negative feedback regulation in the MAPK pathway after MEK inhibition could be a major cause for the lack of clinical efficacy [3, 4]. MAPK signaling was once considered a linear and relatively simple receptor-to-nucleus pathway [31–33]. However, we now know that autoregulatory negative feedback loops in the MAPK pathway precisely control signaling output (Fig. 1) [33]. In cancer, both inhibition and hyperactivation of ERK 1 and ERK2 cause growth inhibition or senescence [34]. Thus, their activity is maintained within a narrow threshold range. For example, ERK1 and ERK2 feedback phosphorylates CRAF and BRAF, which impairs their ability to bind to RAS in the plasma membrane and/or disrupts heterodimeric association of BRAF with CRAF, causing attenuation of RAF protein kinase activity [33]. In contrast, MEK inhibitors reduce the activity of ERK1 and ERK2 and then relieve the feedback inhibition of RAF, resulting in enhancement of RAF kinase activity [35]. Consequently, RAF’s downstream effectors MEK1 and MEK2, and then ERK1 and ERK2, are paradoxically activated. This was recently identified as adaptive drug resistance, a subtype of primary (intrinsic or innate) drug resistance [36]. Targeted therapy inhibits the oncogenic pathway but also relieves the negative feedback [37]. Consequently, the targeted cell signaling is paradoxically activated. This rebound effect appears immediately after exposure of cancer cells to the inhibitor, which enables them to survive showing little primary response [38].

### Second-generation MEK inhibitors are feedback busters

To overcome adaptive drug resistance, second-generation MEK inhibitors, so-called “feedback busters,” were developed: trametinib, GDC-0623, and RO5126766 (CH5126766) [3, 4]. The compounds inhibit not only the ability of MEK1 and MEK2 to elevate ERK1 and ERK2, but also impair the ability of RAF to phosphorylate MEK1 and MEK2 by disrupting the conformation of the activation loop of MEK1 and MEK2. Among them, only trametinib (see above) has been approved. Other MEK inhibitors, including GDC-0623 and RO5126766, require further study to support their safety and efficacy [3, 39, 40]. In BRAF-mutated melanoma, CRAF and RAS activities are diminished by activated BRAF [3, 4]. Thus, the combination of BRAF and MEK inhibitors (dabrafenib and trametinib) is remarkably effective [28–30]. Likewise, although cobimetinib is a first-generation MEK inhibitor, its addition to the BRAF inhibitor vemurafenib significantly increases the durable response rate over single-agent BRAF-inhibitor therapy in patients with BRAF V600-mutated metastatic melanoma [25].

Previous studies have uncovered MEK-independent but kinase-dependent functions of CRAF. For example, CRAF was found directly to phosphorylate and inactivate retinoblastoma protein (RB), leading to cell-cycle progression [41]. Likewise, phosphorylated-CRAF at Ser338 was reported to localize to the mitotic spindle to promote mitosis in cancer cells [42]. Thus, a combination of MEK and CRAF inhibitors may also prevent the non-MAPK pathways downstream of CRAF. Although the biological phenomena are less relevant to RAF protein kinase activation after MEK inhibition, CRAF was reported to have kinase- and MEK-independent functions in cancer cells [43].

### Cyclin D1 plays a critical role as a downstream effector molecule in the MAPK pathway

Cyclin D1 is a major transcriptional target of ERK1 and ERK2. Given the importance of the MAPK signaling pathway for G1 to S cell-cycle transition [11, 12, 44], it could serve as a biomarker to determine the clinical efficacy of MAPK-targeted therapy.

The critical role of cyclin D1 for MAPK-mediated oncogenesis was established first in the murine model for skin cancer [45] and later for breast cancer [46]. In HER2-driven or oncogenic HRAS-driven breast cancer in mice, tumor development was specifically protected by depleting cyclin D1 or CDK4 or expressing the CDK4- or CDK6-specific inhibitor INK4A or a dominant-negative mutant form of K112E cyclin D1 [46–50].

This finding led to a new therapeutic opportunity targeting the cyclin D1-CDK4/6 complex by a CDK4/6 inhibitor, palbociclib (PD0332991) [51–53]. This inhibits proliferation of estrogen receptor (ER)-positive luminal breast cancer cell lines in the presence or absence of HER2 amplification [51]. In fact, ER-positive breast cancer cells highly elevate the activity of ERK1 and ERK2 through HER2- and PKCδ-mediated RAS activation [52]. Consistent with the preclinical study, patients with ER-positive breast cancer showed the best clinical responses to palbociclib in combination with endocrine therapy [54]. Although other clinical trials have shown single-agent activity in mantle-cell lymphoma, liposarcoma, and teratoma with a manageable toxicity profile [53], expanding the use of CDK4/6 inhibitors beyond ER-positive breast cancer is challenging—a circumstance that may be attributable to redundancy in function among different CDKs [11].

Mouse embryonic fibroblasts can proliferate with CDK1 as the sole cell cycle-associated CDK [55, 56]. Likewise, most cell types from CDK4 and CDK6 double-knockout mice proliferate normally [57]. Moreover, D-cyclins can associate with CDK2 to drive G1-S cell-cycle transition [57]. These facts may suggest redundancy among CDKs, and that selection of appropriate target groups for CDK4/6 inhibition relies on successful identification of the tumor type [53]. Thus, tumors that do not depend on CDK4/6 for G1-S transition and/or can rescue CDK4/6 inhibition by the activity of other CDKs may require alternative approaches, including the use of compounds that affect cyclin D1 transcription or protein turnover and combination therapies that target multiple endpoints of cyclin D1 action simultaneously [50].

### ETS1 and ETS2 transcription factors are major downstream effectors of RAS/MAPK signaling and cooperate with AP-1 transcription factor to regulate target genes

Studies with the polyomavirus enhancer region revealed that ETS family transcription factors are major downstream effectors of RAS/MAPK signaling [58]. The RAS/MAPK response sequence in the polyomavirus enhancer region consists of adjacent binding sites for ETS and AP-1 family transcription factors [59–61]. Also, ETS1 or ETS2 cooperates with c-Fos and c-Jun (components of AP-1) to activate transcription from the polyomavirus enhancer domain [62]. Likewise, downstream target genes of RAS/MAPK in humans often have AP-1 DNA binding sequence adjacent to ETS binding sites in the promoter regions. For example, ETS1, which autoregulates its transcript, has ETS and AP-1 binding sites in the *ETS1* promoter regulatory region [63, 64]. Similarly, the minimum 5′ sequence of *CCND1* (encoding cyclin D1) that retains responsiveness to RAS has both ETS and AP-1 binding sites [9, 10, 44].

There are 28 characterized ETS family members in humans [65]. Among them, 21 are phosphorylated by ERK2 in vitro [66], although not all equally. For example, ERG, FLI1, ETV1 and ETV4 are barely expressed in normal epithelial cells and most of RAS-transformed cells [58, 66, 67]. In contrast, ETS1 and ETS2 are particularly important, and their deletion has been shown to inhibit transformation caused by G12V HRAS in mouse embryonic fibroblasts [68]. ETS1 and ETS2 are respectively phosphorylated by ERK1/2 at Thr38 and Thr72; this enhances association with p300 or CREB binding protein (CBP) transcriptional co-activator, resulting in an increase in the transactivational activity of their target genes [69–71]. These observations suggest that ETS1 and ETS2 are major effectors of RAS/MAPK signaling, and thus can be alternative targets for the RAS/MAPK pathway. In support of this, because the inhibition of ERK1/2 by MEK inhibitors triggers an adaptive drug response, we may, by targeting ETS1 and ETS2—the downstream effectors of ERK1/2—avert this limitation.

### ETS1 and ETS2 have both distinct and redundant roles

ETS1 and ETS2 expression is ubiquitous, although cell-type-dependent [67]. ETS1 is expressed at higher levels in the spleen and thymus; ETS2 is elevated in the brain, fetal liver, muscle, and uterus. In the lung, both genes are expressed at the same elevated level. In mice ETS2 gene manipulation causes embryonic death before 8.5 days’ gestation owing to defects in extraembryonic tissue rather than to a major embryonic anomaly [72]. In contrast, ETS1-deficient mice are viable, but demonstrate abnormalities in the differentiation of all lymphoid lineages [73, 74]. These observations suggest that ETS1 and ETS2 have distinct roles.

Likewise, a recent study demonstrated that ETS1, but not ETS2, is necessary for cell migration after RAS/MAPK activation in DU145 prostate cancer cells [58]. Similar observations were reported in ETS1-mediated epithelial cell morphogenesis after activation of MET-RAS-MAPK signaling by hepatocyte growth factor [75].

However, ETS1 and ETS2 depletion synergistically inhibits the RAS-induced cellular transformation in mouse embryonic fibroblast cells [68]. Thus, ETS1 and ETS2 would have a redundant role for the transforming effects of oncogenic RAS.

### Inhibiting or modulating ETS1 transcription factor

Phosphorylated Tyr283 protects ETS1, but not ETS2, from proteolysis, which may allow the development of ETS1-specific small-molecule inhibitors [76, 77].

**Fig. 2:**
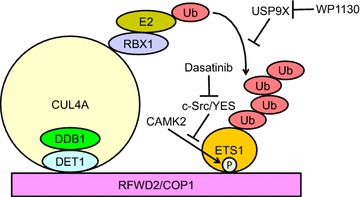
The complex containing RFWD2/COP1 mediates ubiquitination of ETS1. The E3 ubiquitin ligase RFWD2/COP1 recognizes ETS1 in a CAMK2-mediated Ser276 and Ser282 phosphorylation-dependent manner. Src or YES1 phosphorylates ETS1 at Tyr283, which reverses CAMK2-dependent destabilization of ETS1 protein. The deubiquitinase USP9X prevents EST1 ubiquitination and stabilizes the protein. Thus, dasatinib and WP1130 (Src and USP9X inhibitors, respectively) might target ETS1 for proteolysis

ETS1 protein is destabilized after phosphorylation at Ser276 and Ser282 by calcium/calmodulin-dependent protein kinase II (CAMK2) (Fig. 2) [78, 79]. The phosphorylation enhances an association with ring finger and WD repeat domain 2 protein (RFWD2 or COP1), which is an E3 ubiquitin ligase [80]. Subsequently, phosphorylated-ETS1 is degraded through the ubiquitin–proteasome pathway. Conversely, c-Src or YES1 phosphorylates ETS1 at Tyr283, which reverses CAMK2-dependent destabilization of ETS1 protein [79]. Thus, Src inhibitors such as dasatinib and saracatinib may accelerate rapid turnover of ETS1 protein and might prevent transactivation of ETS1 target genes (Fig. 2). WP1130 is a partly selective deubiquitinase inhibitor for USP9X, USP5, USP14, and UCH37 [81]. A recent report demonstrated that USP9X prevents ETS1 ubiquitination and thereby stabilizes the protein [77]. Thus, WP1130 may accelerate ETS1 protein degradation (Fig. 2) and inhibit transactivation of ETS1 target genes.

Targeting the ETS1 interface might be another approach [82]. One of the early attempts was an inhibition of ETS1-DNA binding interaction by the use of oligonucleotides that mimic the ETS1-binding sites [83]. However, the core ETS binding sequence is shared by various ETS family members, and thus this strategy may not be specific for ETS1 inhibition. In contrast, targeting ETS1 protein–protein interaction might be. ETS1 dimerizes itself or other transcription factors [84–88]. Homodimeric interactions through the ETS domain may play a role in cooperative binding to repeated DNA elements [86], whereas heterodimeric interactions might regulate tissue-specific gene expression [87, 88]. Although ETS1 protein–protein Interactions have great therapeutic promise, further studies are warranted to develop small-molecule inhibitors targeting them.

### Targeting transcriptional co-factors of ETS1 and ETS2

**Fig. 3:**
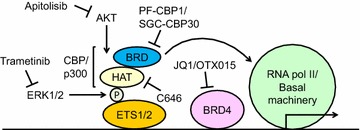
ETS1/2 interacts with CBP/p300 to transactivate target genes. ERK1/2-phosphorylated ETS1 at Thr38 and Ser41, or ETS2 at Thr72 and Ser75, which enhances an association with two closely related transcriptional co-activating proteins, CBP and p300. The binding promotes the assembly of RNA polymerase II and the basal machinery at the initiation of transcription. AKT is likely to activate the ETS1/2-containing transcription factor-enhancer complex by phosphorylating CBP or p300, histone acetyltransferases (HAT) that possess bromodomain (BRD). BRD4 is often co-localized with ETS1 and probably with ETS2 in transcription factor-enhancer complex. Thus, we may inhibit ETS1/2 activity by trametinib (MEK inhibitor), apitolisib (PI3K/mTOR inhibitor), C646 (CBP HAT inhibitor), PF-CBP1 or SGC-CBP30 (CBP BRD inhibitor), or JQ1 or OTX015 (BRD4 inhibitor)

Transcriptional cofactors might represent fascinating therapeutic targets for both ETS1 and ETS2 [76]. These assemble on transcription-binding DNA sequences with transcription factors to influence RNA polymerase II activity (Fig. 3). ERK1/2-phosphorylated ETS1 at Thr38 and Ser41, and ETS2 at Thr72 and Ser75, directly interact with two closely related transcriptional co-activating proteins, CBP and p300 (Fig. 3) [89]. The association promotes the assembly of RNA polymerase II and the basal machinery at the initiation of transcription. Indeed, the recruitment of CBP or p300 enhances transactivation of ETS1 and ETS2 target genes more than 20 times [89]. Thus, the RAS/MAPK signaling pathway activates ETS1 and ETS2 by promoting a unique binding interface with p300 or CBP. Conversely, inhibition of ERK1/2 by a MEK inhibitor disrupts the interface with p300/CBP, decreasing transcriptional activity of ETS1 and ETS2 (Fig. 3).

Likewise, AKT might activate the ETS1- or ETS2-containing transcription factor-enhancer complex by phosphorylating its co-activator p300 or CBP (Fig. 3) [44, 90–94]. In fact, AKT phosphorylates p300 at Ser1834, which is essential for its transcription from the promoter of intercellular adhesion molecule-1 [95], whose transcription is also activated by ETS1 and ETS2 [96, 97]. This possibility was supported by our protein motif analysis [44]. CBP has highly stringent potential AKT phosphorylation sites at Ser381, Ser1733 and Thr1833, all of which are in CBP’s CH1 and CH2/CH3 domains, which interact with ETS1 [98]. Thus, inhibition of AKT might attenuate CBP/p300 activity, resulting in reduction of transactivation of ETS1 and ETS2 target genes (Fig. 3).

CBP and p300 are not only transcriptional co-activators but also histone acetyl-transferases (HAT) that acetylate both histone and non-histone proteins (Fig. 3) [99, 100]. Moreover, they possess bromodomain (BRD), which recognizes acetylated-lysine residues on proteins (Fig. 3) [100]. Thus, if we could decrease the HAT activity of CBP/p300 or perturb BRD-mediated protein–protein interactions, this might prevent chromatin remodeling and ETS1/2 binding to DNA and transcriptional regulatory complexes (Fig. 3). Recently, highly selective small-molecule inhibitors for CBP/p300, including SGC-CBP30 and PF-CBP1, were developed with a structure-based design to inhibit CBP BRD [101, 102].

Likewise, a recent study demonstrated that BRD-containing protein 4 (BRD4) is highly enriched at enhancers associated with genes involved in multiple profibrotic pathways, where BRD4 is co-localized with profibrotic transcription factors, including ETS1, SRF, SMAD3, and NF-κb/p65 (Fig. 3) [103]. Thus, it may be possible to target ETS1/2 by inhibiting BRD4 with small-molecule inhibitors such as JQ1 and OTX015 [104, 105].

### Hormonal therapy may augment ETS-targeted therapy

Given that selection of appropriate target groups for ETS1/2 inhibition relies on successful identification of tumor type, ETS-targeted therapy may have a synergistic benefit with standard therapies.

The transcriptional function of the androgen receptor (AR) is essential for the genesis and development of prostate cancer [106]. A recent report demonstrated that ETS1-binding sequences were specifically enriched in AR-targeted genes [107]. Likewise, estrogen receptor α (ERα) associates directly with ETS1 to stimulate estradiol-dependent growth in breast cancer and neuroblastoma cells [108, 109]. These observations suggest that simultaneous treatments of these cancers with ETS1 and hormonal therapy may enhance clinical outcomes.

### A potential problem of ETS-targeted therapy

As described above, the ETS1-containing transcription factor-enhancer complex could be disrupted with small-molecule inhibitors by specifically targeting ETS1 for proteolysis. However, the redundancy between ETS1 and ETS2 is a possible drawback of an ETS1-specific therapy, as ETS2 might subsequently compensate for ETS1. Likewise, it is possible that ETS1/2-targeted therapy may inhibit autoregulatory negative feedback loops, causing paradoxical activation of ERK1 and ERK2.

We recently studied the mechanism of ERK1/2 activation after EGFR inhibition in non-small cell lung cancer (NSCLC) harboring EGFR mutations [44, 90–94]. Our study demonstrated that EGFR inhibition in lung cancer cells generates a drug-tolerant subpopulation by blocking AKT activity and thus inactivating ETS1 function. The remaining cells enter a dormant, non-dividing state because of the inhibited transactivation of ETS1 target genes, cyclins D1, D3, and E2. Moreover, ETS1 inactivation inhibits transcription of dual specificity phosphatase 6 (DUSP6), a negative regulator specific for ERK1/2. The resulting activation of ERK1/2 combines with c-Src to renew activation of the RAS/MAPK pathway, causing increased cell survival by accelerating BIM protein turnover. By analogy, ETS-targeted therapy may activate ERK1 and ERK2 by inhibiting DUSP6.

However, conflicting data exist regarding the impact of ETS1 inhibition on MAPK signaling. A recent report has shown that ETS1 knockdown in DU145 prostate cancer cells activates dual specificity phosphatase 4 (DUSP4), DUSP6, and sprouty RTK-signaling antagonist 4 (SPRY4) [58]. Because DUSP4/6 and SPRY4 are negative regulators for ERK1/2 and RTK, respectively [31, 61, 110], these observations may suggest that ETS1 is required for robust RAS/ERK pathway activation, and reducing its activity would attenuate the activity of ERK1 and ERK2. This is an area where further study is warranted.

## Conclusions

Efficacy of MEK inhibitors has been unsatisfactory in most tumors, despite successful pre-clinical testing. This fact has prompted us to develop an alternative approach. In this article, we have proposed transcription factor ETS1 and ETS2 as alternate therapeutic targets because they are major effectors of RAS/MAPK signaling. Chemical targeting of ETS1 for proteolysis would be among the few curative strategies in cancer therapeutics. Src and USP9X inhibitors might accomplish this by accelerating ETS1 protein turnover. Likewise, targeting ETS1 interface might have great therapeutic promise because ETS1 dimerizes itself or other transcription factors to regulate transcriptional target genes. Also, transcriptional cofactors of ETS1 and ETS2, including CBP/p300 and BRD4, may represent the other fascinating therapeutic targets around the transcription factor-enhancer complex. However, there exists a potential issue in ETS-targeted therapy. The remedy may inhibit autoregulatory negative feedback loops in the MAPK pathway, which might cause resistance to cell death by paradoxically activating ERK1 and ERK2. Further research is warranted to explore ways to inhibit ETS1 and ETS2 for clinical application.

## Abbreviations

MAPK: mitogen-activated protein kinase
Ser: serine
Thr: threonine
Tyr: tyrosine
ERK1 and ERK2: extracellular signal-regulated kinase 1 and 2
RTK: receptor tyrosine kinase
RB: retinoblastoma protein
ER: estrogen receptor
PKC: protein kinase C
CDK: cyclin-dependent kinase
CBP: CREB binding protein
CAMK2: calcium/calmodulin dependent protein kinase II
RFWD2: ring finger and WD repeat domain 2
HAT: histone acetyltransferase
BRD: bromodomain
BRD4: bromodomain-containing protein 4
NSCLC: non-small cell lung cancer
EGFR: epidermal growth factor receptor
DUSP6: dual specificity phosphatase 6
DUSP4: dual specificity phosphatase 4
SPRY4: sprouty RTK signaling antagonist 4
